# Enorme ostéosarcome tibiale: à propos d'un cas historique

**DOI:** 10.11604/pamj.2015.20.430.6293

**Published:** 2015-04-29

**Authors:** Abderrahman El Mazghi, Khalid Hassouni

**Affiliations:** 1 Faculté de Médecine et de Pharmacie, Université Sidi Mohamed Ben Abdellah, Service de Radiothérapie, CHU Hassan II, Fès, Maroc

**Keywords:** Ostéosarcome, tibiale, négligé, Osteosarcoma, tibial, neglected

## Image en medicine

L'ostéosarcome est la plus fréquente des tumeurs malignes primitives de l'os avec une incidence annuelle de 4,8 par million d'habitants de moins de 20 ans. Soixante pour cent d'entre eux surviennent chez les enfants âgés de 10 à 20 ans. Le traitement chirurgical radical était le traitement de l'ostéosarcome avant les années 1970. Il n'entraînait que 10 à 15% de survie à cinq ans. Depuis la découverte dans les années 1970 de la chimio-sensibilité de cette tumeur, le traitement de l'ostéosarcome a complètement changé. Les progrès de la chimiothérapie et de la chirurgie conservatrice ont contribué à diminuer les indications de la radiothérapie d'autant plus que l'ostéosarcome est décrit de longue date comme radio-résistant, avec pour corollaire la nécessité de délivrer de hautes doses d'irradiation supérieures à 60 Gy avec un fractionnement et un étalement classiques, pour permettre la stérilisation tumorale. La radiothérapie externe peut cependant être discutée en tant que traitement local à visée curative des tumeurs inopérables ou en tant que traitement adjuvant post-chirurgical dans certains cas. Nous rapportons le cas d'une jeune patiente de 18 ans, sans antécédent particulier, qui présentait un ostéosarcome conventionnel de haut grade du tiers supérieur de la jambe droite négligé, de 25 cm de grand axe, non métastatique sur le scanner thoraco-abdomino-pelvien et la scintigraphie osseuse. La patiente est décédée après la première cure de chimiothérapie et avant tout acte chirurgical.

**Figure 1:**
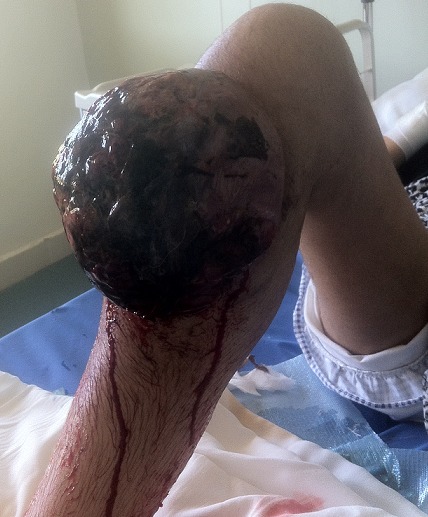
énorme masse tumorale, hémorragique du tiers supérieur de la jambe droite

